# Supplementary material for: Hygrothermal simulation data of a living wall system for decentralized greywater treatment
Source: Data Brief. 2021 Dec 27;40:107741. doi: 10.1016/j.dib.2021.107741 (PMC8741426; doi:10.1016/j.dib.2021.107741)
Supplement: Supplementary file 1 [file mmc1.zip › DataInBrief_Submission/Alsaad et al._Data in brief.docx]

**Article information**

**Article title**

Hygrothermal simulation data of a living wall system for decentralized greywater treatment

**Authors**

Hayder Alsaad*, Maria Hartmann, Conrad Voelker

**Affiliations**

Bauhaus-University Weimar, Department of Building Physics, Weimar, Germany

**Corresponding author’s email address and Twitter handle**

[hayder.alsaad@uni-weimar.de](mailto:hayder.alsaad@uni-weimar.de)

**Keywords**

Coupling, ENVI-Met, Delphin, Heat transport, Moisture transport

**Abstract**

This dataset presents the numerical analysis of the heat and moisture transport through a facade equipped with a living wall system designated for greywater treatment. While such greening systems provide many environmental benefits, they involve pumping large quantities of water onto the wall assembly, which can increase the risk of moisture in the wall as well as impaired energetic performance due to increased thermal conductivity with increased moisture content in the building materials. This dataset was acquired through numerical simulation using the coupling of two simulation tools, namely Envi-Met and Delphin. This coupling was used to include the complex role the plants play in shaping the near-wall environmental parameters in the hygrothermal simulations. Four different wall assemblies were investigated, each assembly was assessed twice: with and without the living wall. The presented data include the input and output parameters of the simulations, which were presented in the co-submitted article [1].

**Specifications table**

| **Subject** | Civil and Structural Engineering |
| --- | --- |
| **Specific subject area** | Heat and moisture transport through a facade equipped with a living wall system designated for greywater treatment |
| **Type of data** | Tables  Figures  Comma-separated values (CSV) files |
| **How the data were acquired** | Numerical simulations using Envi-Met (version 4.4) [2] and Delphin (version 6) [3]. Envi-Met was used to simulate the effect of vegetation on the local climatic parameters at the living wall. Subsequently, Delphin was used to conduct the hygrothermal simulations using the local parameters calculated by Envi-Met. |
| **Data format** | Raw, formatted |
| **Description of data collection** | Four wall assemblies were simulated: a brick wall, a precast plate, a limestone wall, and a double-shell wall, which are commonly used in Germany [4,5]. The local exterior boundary conditions were acquired from Envi-Met, which utilized the weather data provided by the German weather service. The interior boundary conditions corresponded to the model defined by the DIN EN 15026 [6] and the WTA [7]. |
| **Data source location** | Institution: Bauhaus-University Weimar  City/Town/Region: Weimar  Country: Germany  Latitude and longitude: 50°59'0"N - 11°19'0"E |
| **Data accessibility** | With the article |
| **Related research article** | H. Alsaad, M. Hartmann, C. Voelker, The effect of a living wall system designated for greywater treatment on the hygrothermal performance of the facade, Energy and Buildings (2022) 111711. https://doi.org/10.1016/j.enbuild.2021.111711. |

**Value of the data**

- This dataset illustrates how different wall assemblies can have different reactions to facade greening systems. It signifies the impact of correct selection of building material and insulation strategies when planning to implement such systems.
- The methodology and dataset presented in this article allow other researchers and building physicists to conduct further simulations using diverse wall assemblies, geometries, and boundary conditions.
- The presented input data can be used as boundary conditions in other studies that target the same topic.
- The presented output data allow for further analysis of the impact of novel decentralized greywater treatment systems on the facade

**Data description**

This article presents the full dataset of the conducted numerical simulations. While the supported article presented analysis, discussions, and insights into the data, it was not possible to present all the data there due to length limitations. Therefore, the supported article presented filtered data illustrating summer/winter and day/night cycles. Summer and winter seasons were defined according to summer and winter solstices in the northern hemisphere; day and night hours were defined as the shortest day or night period within the season in question at the simulation location (Mannheim, Germany). In the present article, however, the simulated values are presented throughout the whole course of the year.

In Figures 1 through 8, a comparison between a bare facade (reference wall, “no greening”) and a facade equipped with the investigated living wall (“with greening”) is presented. These figures constitute the output acquired from the Delphin models. In all figures, orange data series correspond to the bare facade while green data series represent the same facade when covered with the living wall. Each figure consists of four sub-diagrams corresponding to the four investigated wall assemblies. More information about these walls is presented in the following section (Experimental design, materials and methods). Figures 1 and 2 present the exterior and interior surface temperature of the investigated walls in °C, respectively. Figure 3 shows the simulated relative humidity of the exterior surface of the investigated wall assemblies in %. In these Figures, the surface corresponds to the finished surface of the wall. This means that the exterior surface of the greened variation represents the wall surface behind the living wall system. Figure 4 illustrates the simulated heat flux through the investigated wall assemblies in W/m^2^. These data were exported from the Delphin models as the heat flow over the inner boundary cell. The positive values refer to heat loss from the indoor air towards the outdoors. Conversely, negative values indicate heat flow from the outdoors to the indoors. Figure 5 presents the simulated moisture content in the investigated wall assemblies in kg. These values correspond to the total mass density of liquid water and water vapour in the wall. Figure 6 shows the simulated degree of saturation at the exterior finishes of the investigated wall assemblies in %. This corresponds to the degree of saturation of lime plaster in the cases of the brick wall and the limestone wall, concrete in the case of the precast concrete plate, and veneer brick in the case of the double-shell wall. The presented values of the degree of saturation indicate the percentage of pore space filled with liquid water. Finally, Figures 7 and 8 present air temperature and relative humidity adjacent to the wall, respectively. This corresponds to the values directly in front of the wall in the ‘no greening’ cases and in the air gap in the ‘with greening’ cases.

The raw data of these diagrams are attached to this article as comma-separated values (CSV) files. Each file name starts with ‘Output’ followed by ‘Delphin’ to indicate that the file contains simulation results from Delphin. Afterwards, the presented parameter is mentioned briefly in the file name; an example for the names of the attached results files is “Output_Delphin_Heat flux.” Further details about the presented parameters and their units are presented in the first line of the CSV file. Moreover, an additional results file named “Results_U-values” is attached to document the calculated U-values of the investigated wall assemblies. The calculation methods are presented in the next section. Besides results and output files, the input files used for the simulations are also attached to this article to allow re-conducting the simulations. The names of the input files follow the same logic used for naming the output files. The input files include the exterior boundary conditions (i.e. weather file) used for the Envi-Met simulations and the exterior boundary conditions used for the Delphin models (both ‘with greening’ and ‘no greening’ models). It is important to note that the exterior boundary conditions used for Delphin correspond to the local climatic conditions acquired through the Envi-Met model. Further attached Delphin input files are the greywater supply profile assigned to the substrate of the living wall and the interior boundary conditions which correspond to the adaptive indoor climate model defined by the DIN EN 15026 [6] and the Association for Science and Technology of Building Maintenance and Monuments Preservation (WTA) [7]. This model calculates the daily mean indoor temperature and relative humidity based on the value of the daily mean outdoor temperature. The so-called Normal+5% model for the indoor air humidity was utilized, which includes a safety margin of an additional 5% to the indoor relative humidity to accommodate local increases in humidity derived from specific functions of the room adjacent to the facade (e.g. a bathroom).


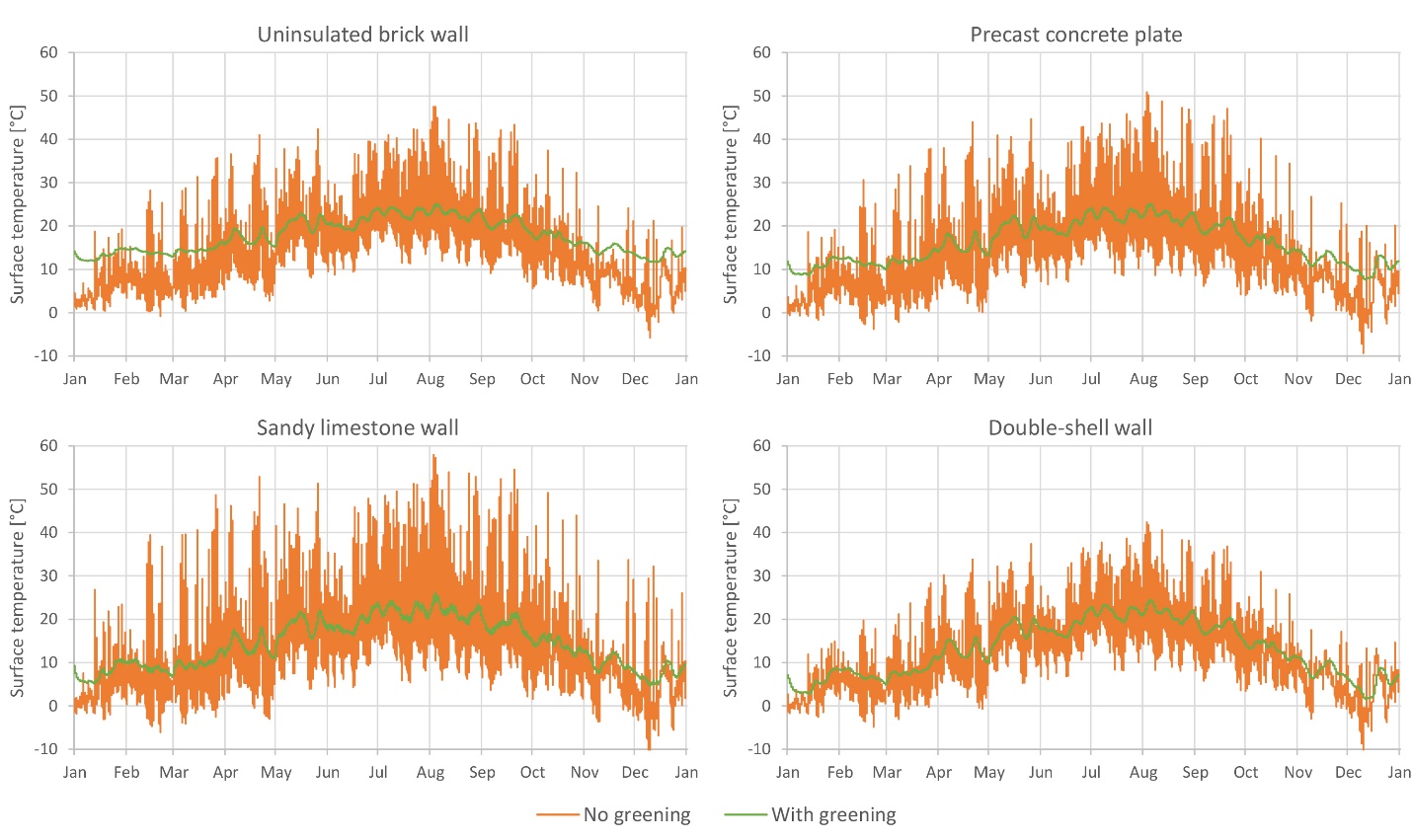


Figure 1 The simulated exterior surface temperature of the investigated wall assemblies


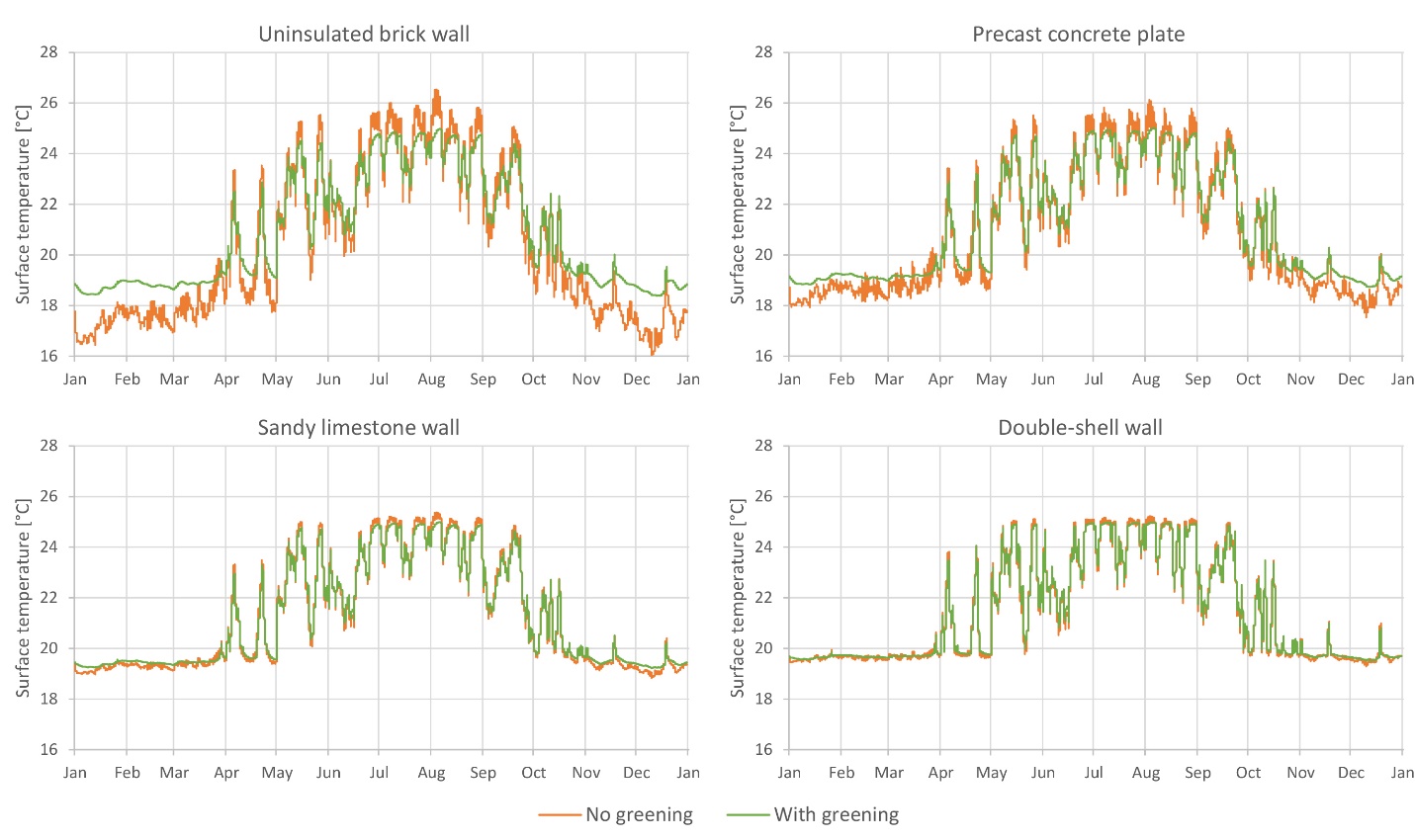


Figure 2 The simulated interior surface temperature of the investigated wall assemblies


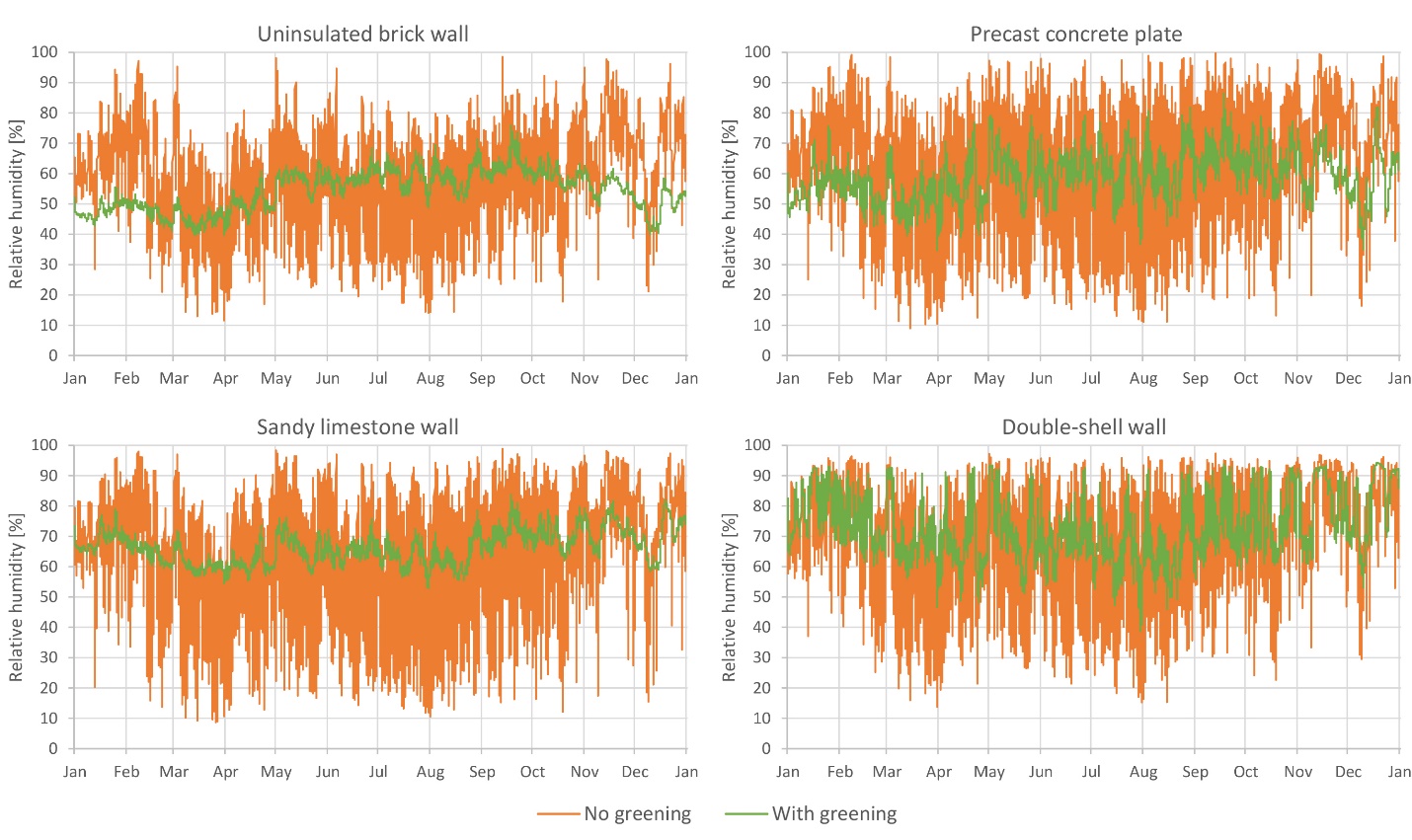


Figure 3 The simulated relative humidity of the exterior surface of the investigated wall assemblies


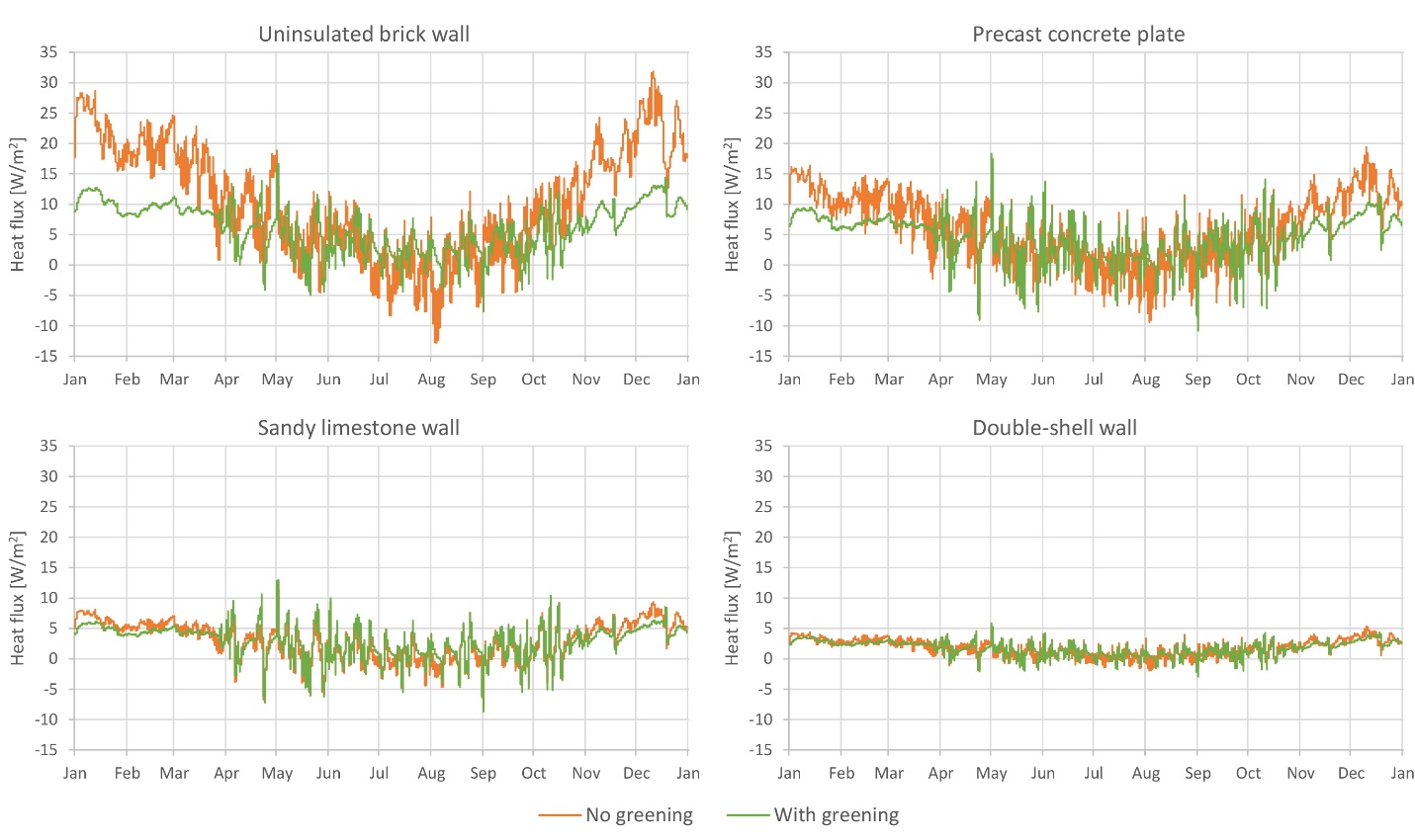


Figure 4 The simulated heat flux through the investigated wall assemblies


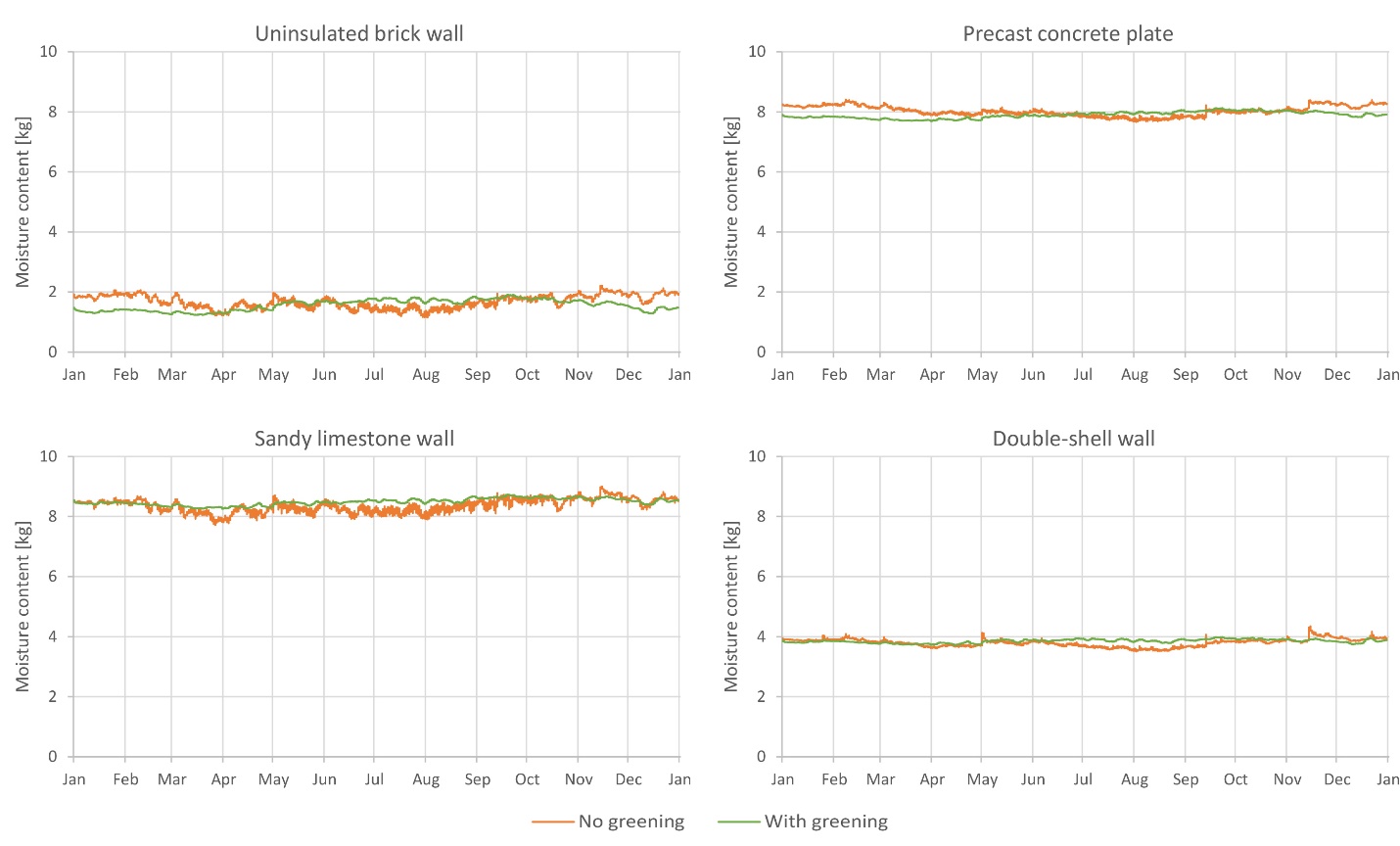


Figure 5 The simulated moisture content in the investigated wall assemblies


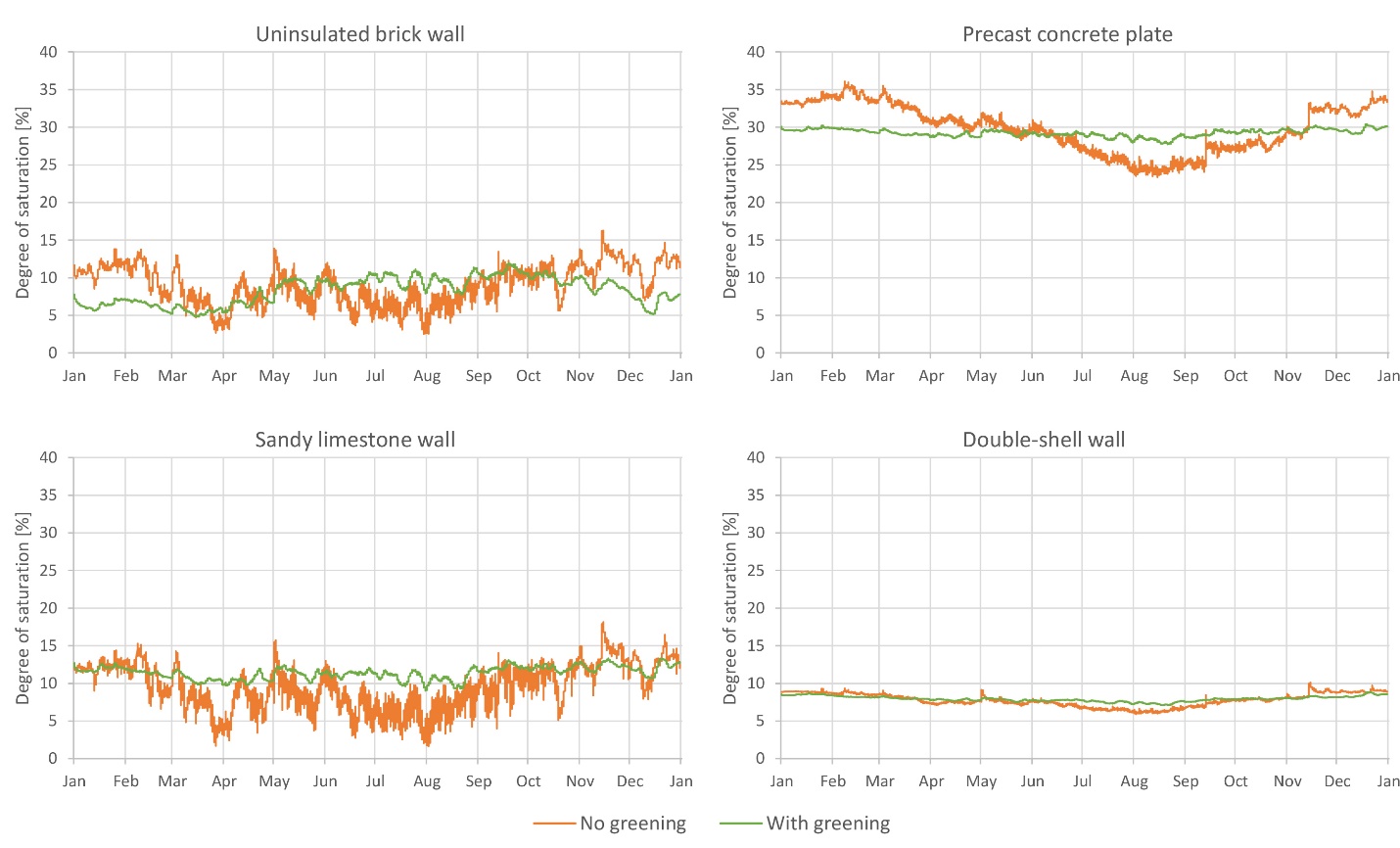


Figure 6 The simulated degree of saturation at the exterior finishes of the investigated wall assemblies


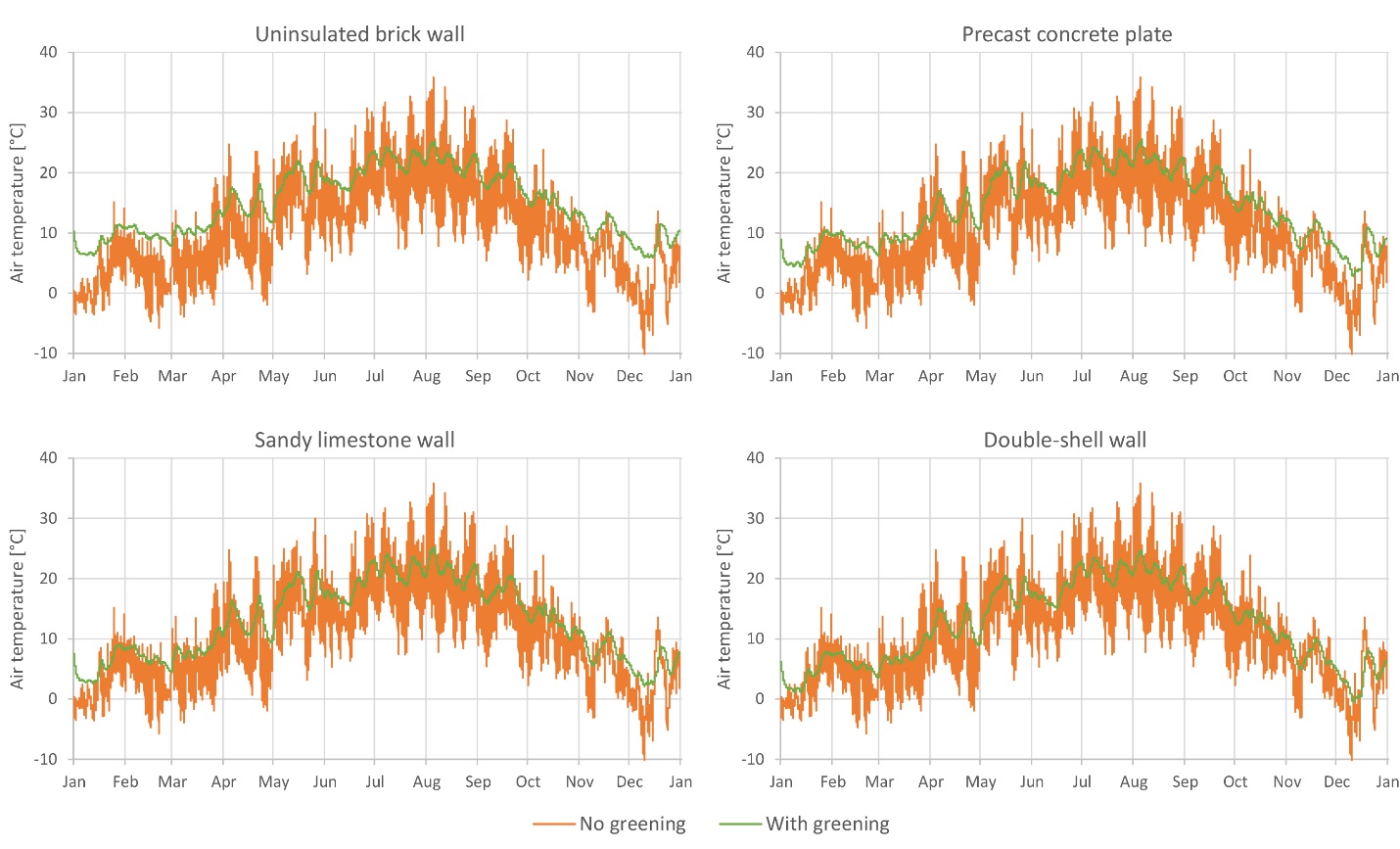


Figure 7 The simulated air temperature adjacent to the wall


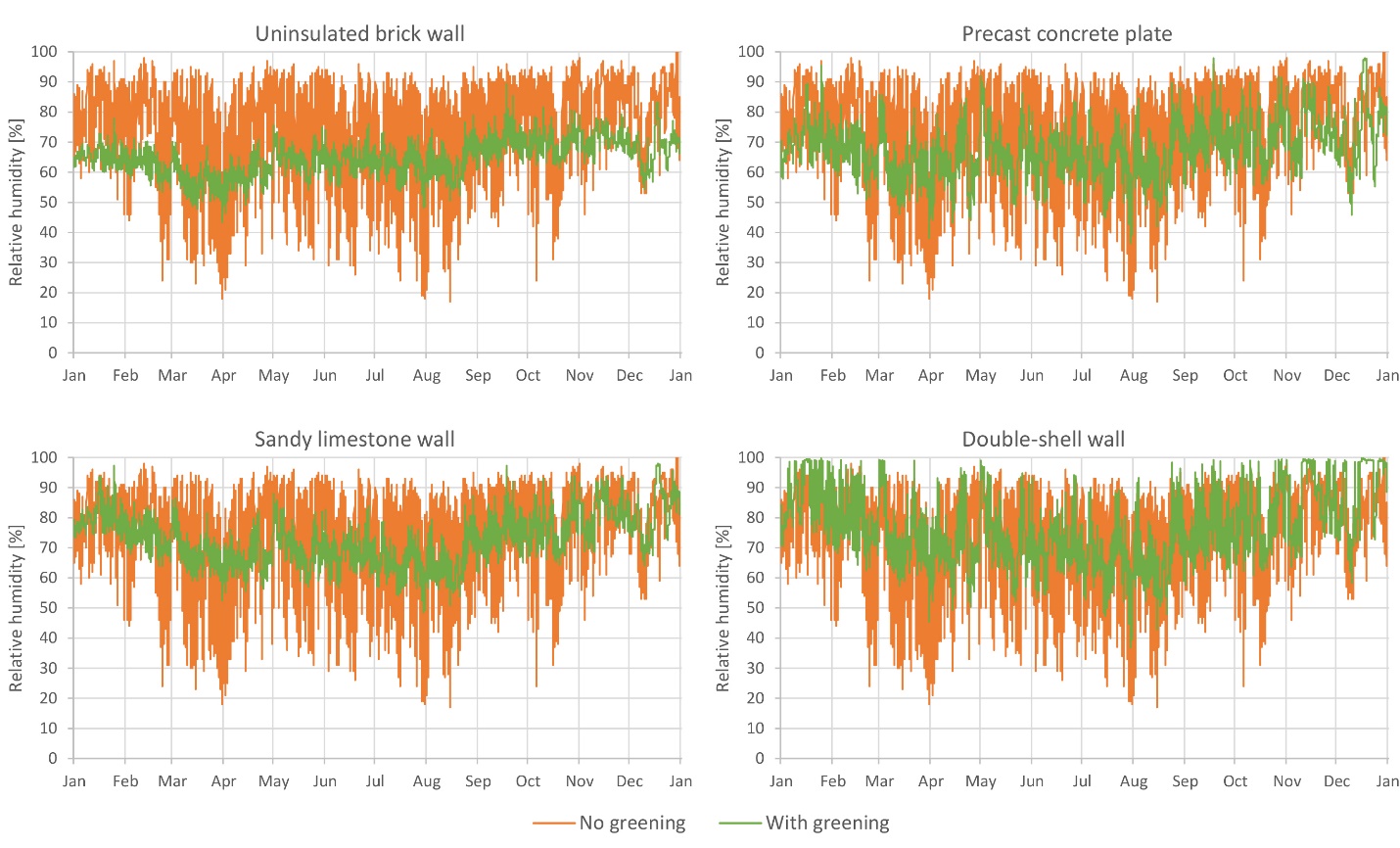


Figure 8 The simulated relative humidity adjacent to the wall

**Experimental design, materials and methods**

The simulation data were acquired using the coupling of Envi-Met and Delphin [8]. The findings of this study are derived from the hygrothermal simulations conducted using Delphin. However, since the complex impact the plants have on the environmental parameters cannot be simulated using Delphin, Envi-Met was used to determine the influence of vegetation on the local climate at the facade. Afterwards, the simulated local climate conditions were imposed as exterior boundary conditions in Delphin.


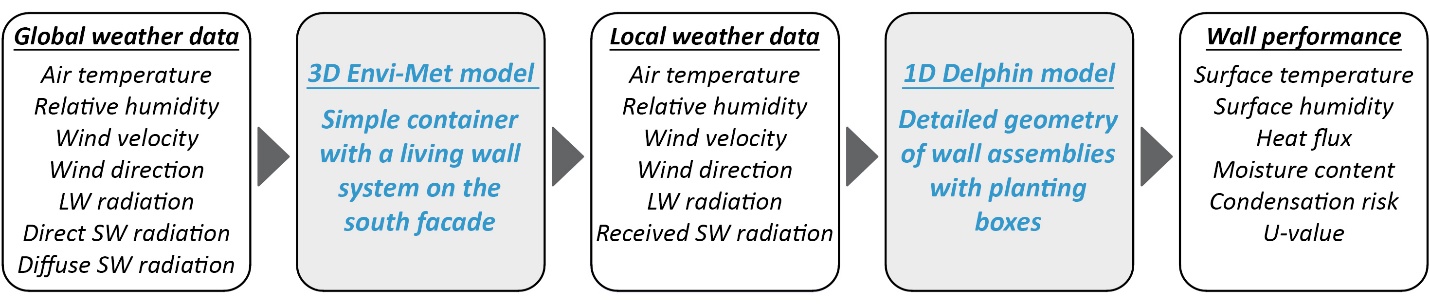


Figure 9 The coupling of Envi-Met and Delphin (modified from [1])

As shown in Figure 9, the first step of the coupling was running the Envi-Met simulations using the global weather data specific to the simulation location. Before conducting the simulations, the different components of the model were separately prepared using the different tools included within Envi-Met. Two models were created: with and without greening. The modelling started with creating the geometry, which was conducted using the modelling tool Spaces. This tool allows setting the domain with the orthogonal Arakawa C-grid and subsequently assigning buildings and trees to grid cells with the desired dimensions and materials, which are adopted from the Database Manager. The full details about the geometry and the other settings implemented in Envi-Met are reported in Table 1. Once the geometry was ready, the Forcing Manager was used to generate the full-forcing file using the weather data. The full-forcing file included air temperature, velocity, relative humidity, wind direction, longwave radiation, and shortwave radiation (direct and diffuse). The driving rain was not simulated in Envi-Met. Afterwards, the tool Envi-Guide was used to generate the simulation files, define the simulation duration, and set the weather forcing details. The mode ‘Intermediate’ was implemented in this study since no advanced settings were needed, e.g. pollutant concentrations or adjusted output. To avoid possible crashes in the simulations, separate simulation files were generated for each month. Thus, a total of 24 simulation files were created (12 months with greening and 12 months without greening). The simulation files were imported into the solver Envi-Core to run the simulations; the output data were processed using the post-processer Leonardo. Microsoft Excel was used subsequently to combine the output of all months into a single local weather file, one for the ‘with greening’ case and a second for the ‘no greening’ case. The parameters comprising the local weather data are air temperature, wind speed, and relative humidity in the foliage of the living wall, the total short-wave and long-wave radiation received by the surface behind the foliage, and wind direction in front of the living wall.

After the Envi-Met simulations, the second step of the coupling, namely the Delphin simulations, were conducted. The modelling process started by creating the geometries, assigning materials, and generating the grid. The details of the Delphin models are shown in Table 2. Afterwards, the local weather data resulting from Envi-Met were used as boundary conditions in Delphin. This was achieved by creating separate *.ccd boundary conditions files for each parameter in the local weather data. These *.ccd files were then imposed on the exterior boundaries of the geometries using the so-called detailed/scientific interface. Necessary adjustments to the weather file (e.g. setting the driving rain in the ‘with greening’ case to zero) were conducted using the CCMEditor 0.4. Moreover, an additional *.ccd file was created to define the supply profile of greywater in the substrate (see the attached CSV file ‘Input_Delphin_Greywater flow rate’). This file was then assigned as a source profile to the substrate grid cells. Further defined sources included an air change rate and a radiative exchange source in the air gap behind the greening system (Table 2). Moreover, a contact condition was created between the wall and the air gap was defined to set the convective heat transfer coefficient according to the EN ISO 6946 [10]. After conducting the Delphin simulations, Microsoft Excel was used to analyse the impact of facade greening of different hygrothermal parameters (Figures 1 to 8). Moreover, the Delphin simulations allowed calculating the effective thermal transmittance (U-value) of the investigated wall assemblies according to:

|  | $U_{\mathrm{eff}}= \frac{\sum_{j=1}^{n} q}{\sum_{j=1}^{n} (\theta_{i,j}-\theta_{e,j})}$ | (3) |
| --- | --- | --- |

Where q is the heat flux [W/m^2^] and θ_i_ and θ_e_ are the indoor and outdoor air temperatures, respectively [°C]. To acquire a quasi-steady state range, the data filtration proposed by Tudiwer et al. [9] (Data pool B criteria) was conducted using the filtering functions in Microsoft Excel. In addition to calculating the effective U-value from the simulation results, the U-value of each simulated assembly was calculated using the calculation method reported in the standard DIN EN ISO 6946 [10] using the equation:

|  | $U=\left( R_{si}+\sum_{j=1}^{n} \frac{d_{j}}{\lambda_{j}}{+ R}_{se} \right)^{-1}$ | (4) |
| --- | --- | --- |

Where d [m] and λ [W/mK] are the thickness and thermal conductivity of each layer in the wall assembly, respectively. The R_si_ and R_se_ are the interior and exterior surface resistance [m^2^K/W], respectively, which were set according to the values reported in the standard DIN EN ISO 6946 [10].

Table 1 The details and settings of the Envi-Met models

| Software and version | Envi-Met 4.4 |
| --- | --- |
| Type of modelling | 3D |
| Computation domain | 22.5 x 22.5 x 20 m |
| Basic cell size | 0.75 x 0.5 x 0.75 m (dx, dy, dz) |
| Mesh growth rate | 20% on the vertical direction |
| Total number of cells | 20250 |
| Simulated building size | 4.5 x 4.5 x 4.5 m |
| Number of simulated models | Two models; with and without greening |
| Wall assembly | Generic wall construction (d = 410 mm) |
| Living wall area | 4.5 x 3 m |
| Living wall thickness | Plants thickness: 30 cm  Depth of substrate container: 25 cm  Air gap behind substrate container: 5 cm |
| Living wall orientation | South |
| Substrate containers details | Backside: rigid 12 mm polyethylene (PE) plate  Substrate: a mixture of expanded clay aggregate (66.6%) and biochar (33.3%)  Front side: flexible cotton-based textile (neglected in the model) |
| Plants | Albedo: 0.3  Transmittance index: 0.2  Leaf angle distribution: 0.25  Leaf area density: 6 m^2^/m^3^  Leaf area density profile: in summer months 50% higher than in winter months |
| Simulation site | Mannheim, Germany, 49°29'16"N - 08°27'58"E |
| Weather data | Test reference year (TRY) from the German weather service (DWD) |
| Lateral boundaries setup | Full forcing; 30-minute time steps at inflow |
| Indoor conditions | Variable (depending on outdoor conditions) |
| Simulation time step | 2 s at the initialization  1 s throughout the rest of the simulation time |
| Simulation time span | 1 year; hourly data output |
| Turbulence model | E-epsilon 1.5 order turbulence closure |

Table 2 The details and settings of the Delphin models

| Software and version | Delphin 6 |
| --- | --- |
| Type of modelling | 1D |
| Computation domain | 1 m long, depth depended on wall assembly |
| Basic cell size | Minimum size: 1 mm  Maximum size: 50 mm |
| Mesh growth rate | 30% |
| Number of simulated models | Eight models; with and without greening x four wall assemblies: an uninsulated brick wall, a precast concrete building plate, a sandy limestone wall, and a double-shell wall |
| Wall assembly | Assembly details and building materials are presented in Table 3 |
| Wall orientation | South |
| Living wall dimensions | Depth of substrate container: 25 cm  Air gap behind substrate container: 5 cm |
| Substrate containers details | Backside: rigid 12 mm polyethylene (PE) plate  Substrate: a mixture of expanded clay aggregate (66.6%) and biochar (33.3%)  Front side: flexible cotton-based textile (implicitly simulated as an extra vapour diffusion thickness (s_d_-value) of 0.1 m) |
| Simulation site | Mannheim, Germany 49°29'16"N - 08°27'58"E |
| Weather data | Local climatic data acquired from the Envi-Met models |
| Exterior convective heat transfer coefficient | Variable; *h_c_ = h_c0_ + k_h_ · v^kexp^* (1)  Where h_c0_ is the transfer coefficient for still air [W/m^2^K], k_h_ is the slope coefficient for moving air [J/m^3^K], and k_exp_ is the exponent for moving air [-]. |
| Exterior water vapour exchange coefficient | Variable; *ß* = *ß_0_* + *k_v_* · *v^kexp^* (2)  Where *ß_0_* is the exchange coefficient for still air [s/m], *k_v_* is the slope coefficient for moving air [s^2^/m^2^] and *k_exp_* is the exponent for moving air [-]. |
| Exterior short-wave radiation | Absorption coefficient (no greening): 0.6  Absorption coefficient (with greening): 0.4 |
| Exterior long-wave radiation | Emission coefficient (both cases): 0.9 |
| Driving rain | No greening: values adopted from weather data  With greening: zero |
| Indoor conditions | Variable; adaptive indoor climate model based on the value of the daily mean outdoor temperature (Normal+5% model) |
| Indoor heat transfer coefficient | 8 W/m^2^K |
| Indoor surface vapour diffusion coefficient | 2.5e-08 s/m |
| Air change rate in the air gap | highly-ventilated; 20/h |
| Air temperature and humidity in the air gap | Equivalent to the hourly values of the local climate parameters |
| Emission coefficient in the air gap | 0.9 (on both sides) |
| Convective heat transfer coefficient in the air gap | 2.5 W/m^2^K |
| Water vapour exchange coefficient in the air gap | 1.53e-08 s/m |
| Greywater flow rate in the substrate | 75 L/d; water was supplied during the first minute of each hour except at 10:00 pm, 00:00 am, and 02:00 am |
| Initial conditions | Temperature: 20°C  Relative humidity: 80% |
| Simulation time step | 5 s |
| Simulation time span | 4 year; hourly data output; only results from the fourth year was used for the analysis to ensure reaching equilibrium moisture content |
| Solver tolerance | Relative tolerance: 1e-04  Absolute tolerance: 1e-06 |

Table 3 Wall assembly details and building materials used in the Delphin models (modified from [1]). The layers are listed from the interior to the exterior layers.

| **Material** | **Thickness *d* [mm]** | **Density *ρ* [kg/m^3^]** | **Porosity *ϕ* [m^3^/m^3^]** | **Vapour resistance *µ* [-]** | **Heat capacity *c* [J/kgK]** | **Conductivity *λ* [W/mK]** | **Water uptake *Aw* [kg/m^2^s^0.5^]** |
| --- | --- | --- | --- | --- | --- | --- | --- |
| Uninsulated brick wall | | | | | | | |
| Gypsum plaster | 15 | 1043 | 0.606 | 11.3 | 1047 | 0.26 | 0.366961 |
| Full bricks | 380 | 1790 | 0.360 | 14 | 868 | 0.87 | 0.227000 |
| Lime plaster | 15 | 1270 | 0.500 | 12 | 960 | 0.55 | 0.009300 |
| Precast concrete plate | | | | | | | |
| Concrete | 150 | 2320 | 0.143 | 63 | 850 | 2.10 | 0.008333 |
| EPS | 40 | 35 | 0.935 | 50 | 1500 | 0.04 | 0.000010 |
| Concrete | 60 | 2320 | 0.143 | 63 | 850 | 2.10 | 0.008333 |
| Sandy limestone wall | | | | | | | |
| Gypsum plaster | 15 | 1043 | 0.606 | 11.3 | 1047 | 0.26 | 0.366961 |
| Sandy limestone | 240 | 1744 | 0.359 | 27.9 | 850 | 0.82 | 0.049673 |
| EPS | 80 | 35 | 0.935 | 50 | 1500 | 0.04 | 0.000010 |
| Lime plaster | 15 | 1270 | 0.500 | 12 | 960 | 0.55 | 0.009300 |
| Double-shell wall | | | | | | | |
| Gypsum plaster | 15 | 1043 | 0.606 | 11.3 | 1047 | 0.26 | 0.366961 |
| Porous concrete | 175 | 415 | 0.832 | 8.9 | 850 | 0.10 | 0.039065 |
| Mineral wool | 100 | 37 | 0.920 | 1 | 840 | 0.03 | 0.000001 |
| Veneer bricks | 90 | 1852 | 0.301 | 27.1 | 810 | 0.68 | 0.040674 |

**Ethics statements**

No ethical issues are associated with this work.

**CRediT author statement**

**Hayder Alsaad**: Conceptualization, Methodology, Software, Writing - Original Draft, Writing - Review & Editing, Visualization, Project Administration. **Maria Hartmann**: Methodology, Investigation. **Conrad Voelker**: Writing - Review & Editing, Resources, Supervision, Funding acquisition.

**Acknowledgments**

This study was conducted as part of the research project VertiKKA (Vertikale Klimakläranlage zur Steigerung der Ressourceneffizienz und Lebensqualität in urbanen Räumen) funded by the German Federal Ministry of Education and Research (BMBF) as part of the funding initiative Resource-Efficient Urban Districts (RES:Z), Grant No. 033W108G. Their support is appreciated and cherished. We would also like to thank Bjoernsen Beratende Ingenieure GmbH for coordinating the project and the project research partners for their dedication and enthusiasm*.*

# Declaration of interests

The authors declare that they have no known competing financial interests or personal relationships that could have appeared to influence the work reported in this paper.

The authors declare the following financial interests/personal relationships which may be considered as potential competing interests:

References

[1] H. Alsaad, M. Hartmann, C. Voelker, The effect of a living wall system designated for greywater treatment on the hygrothermal performance of the facade, Energy Build. (2022) 111711. https://doi.org/10.1016/j.enbuild.2021.111711.

[2] ENVI-Met, ENVI-Met 4: A holistic microclimate model. [June 18, 2021]; Available from: www.envi-met.com.

[3] J. Grunewald, Documentation of the Numerical Simulation Program DIM3.1", Volume 2: User's Guide. Institute of Building Climatology, Faculty of Architecture, Univesity of Technology Dresden (2000).

[4] Loga T, Stein B, Diefenbach N, Born R, Deutsche Wohngebäudetypologie: Beispielhafte Maßnahmen zur Verbesserung der Energieeffizienz von typischen Wohngebäuden. IWU: Institut Wohnen und Umwelt, Darmstadt, Germany; 2015.

[5] ZUB, Erfassung regionaltypischer Materialien im Gebäudebestand mit Bezug auf die Baualtersklasse und Ableitung typischer Bauteilaufbauten. Zentrum für Umweltbewusstes Bauen e.V; 2009.

[6] DIN EN 15026. Wärme- und feuchtetechnisches Verhalten von Bauteilen und Bauelementen – Bewertung der Feuchteübertragung durch numerische Simulation; German version EN 15026:2007 (Hygrothermal performance of building components and building elements - Assessment of moisture transfer by numerical simulation). Deutsches Institut für Normung e. V; 2007.

[7] WTA. Merkblatt 6-2: Simulation wärme- und feuchtetechnischer Prozesse (Simulation of heat and moisture transfer). Wissenschaftlich-Technische Arbeitsgemeinschaft für Bauwerkserhaltung und Denkmalpflege e.V; 2014.

[8] H. Alsaad, C. Voelker, Heat and moisture transport through a living wall system designated for greywater treatment, Proceedings of BS2021: 17th Conference of the International Building Performance Simulation Association, Bruges, Belgium (2021).

[9] D. Tudiwer, A. Korjenic, The effect of living wall systems on the thermal resistance of the façade, Energy Build. 135 (2017) 10–9. https://doi.org/10.1016/j.enbuild.2016.11.023.

[10] DIN EN ISO 6946. Bauteile - Wärmedurchlasswiderstand und Wärmedurchgangskoeffizient - Berechnungsverfahren; German version EN ISO 6946:2017 (Building components and building elements - Thermal resistance and thermal transmittance – Calculation methods). Deutsches Institut für Normung e. V; 2017.
