# Supplementary figures and images for: Hygrothermal simulation data of a living wall system for decentralized greywater treatment
Source: Data Brief. 2021 Dec 27;40:107741. doi: 10.1016/j.dib.2021.107741 (PMC8741426; doi:10.1016/j.dib.2021.107741)

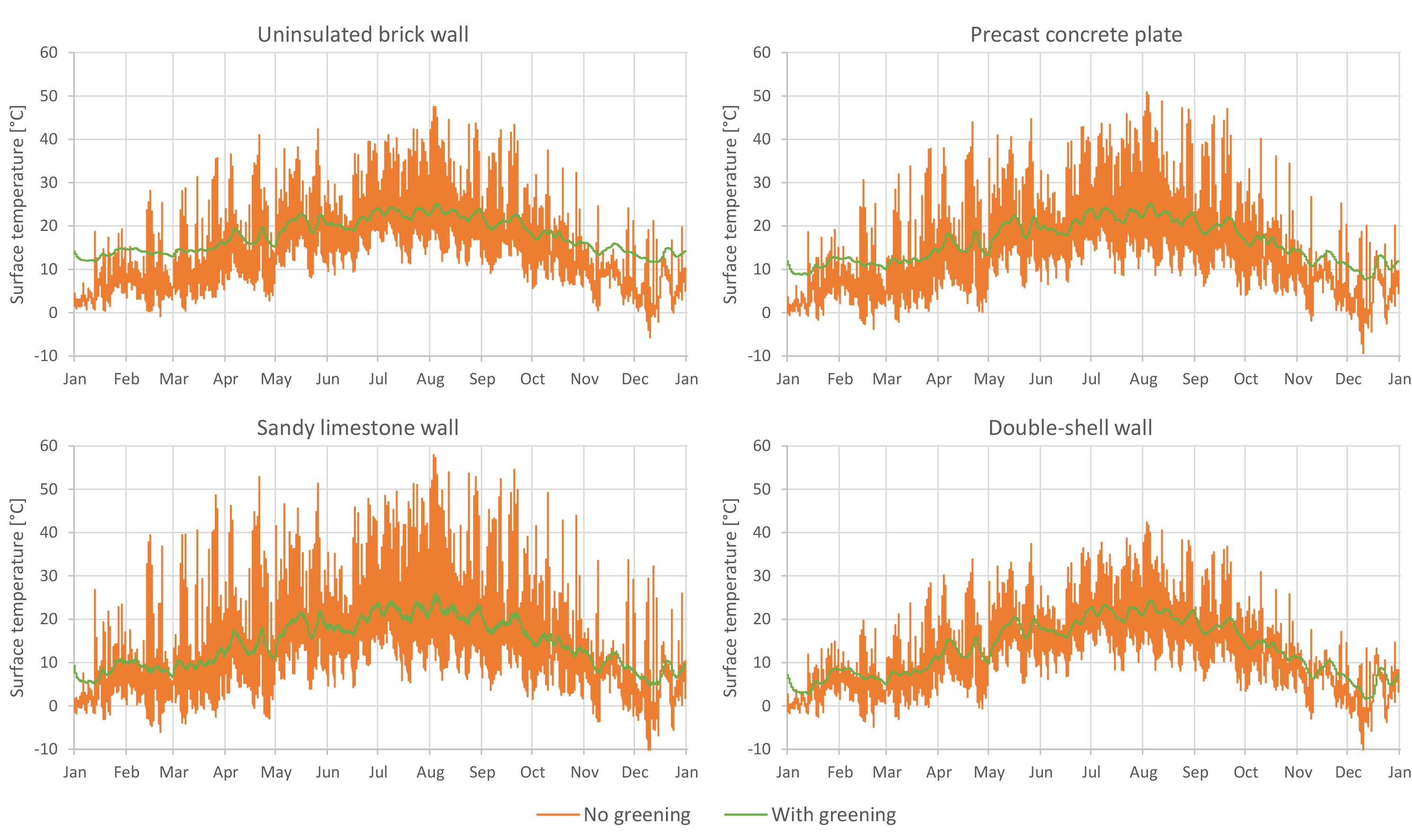

Supplement: Supplementary file 1 [file mmc1.zip › DataInBrief_Submission/Figure 1 The simulated exterior surface temperature of the investigated wall assemblies.jpg]

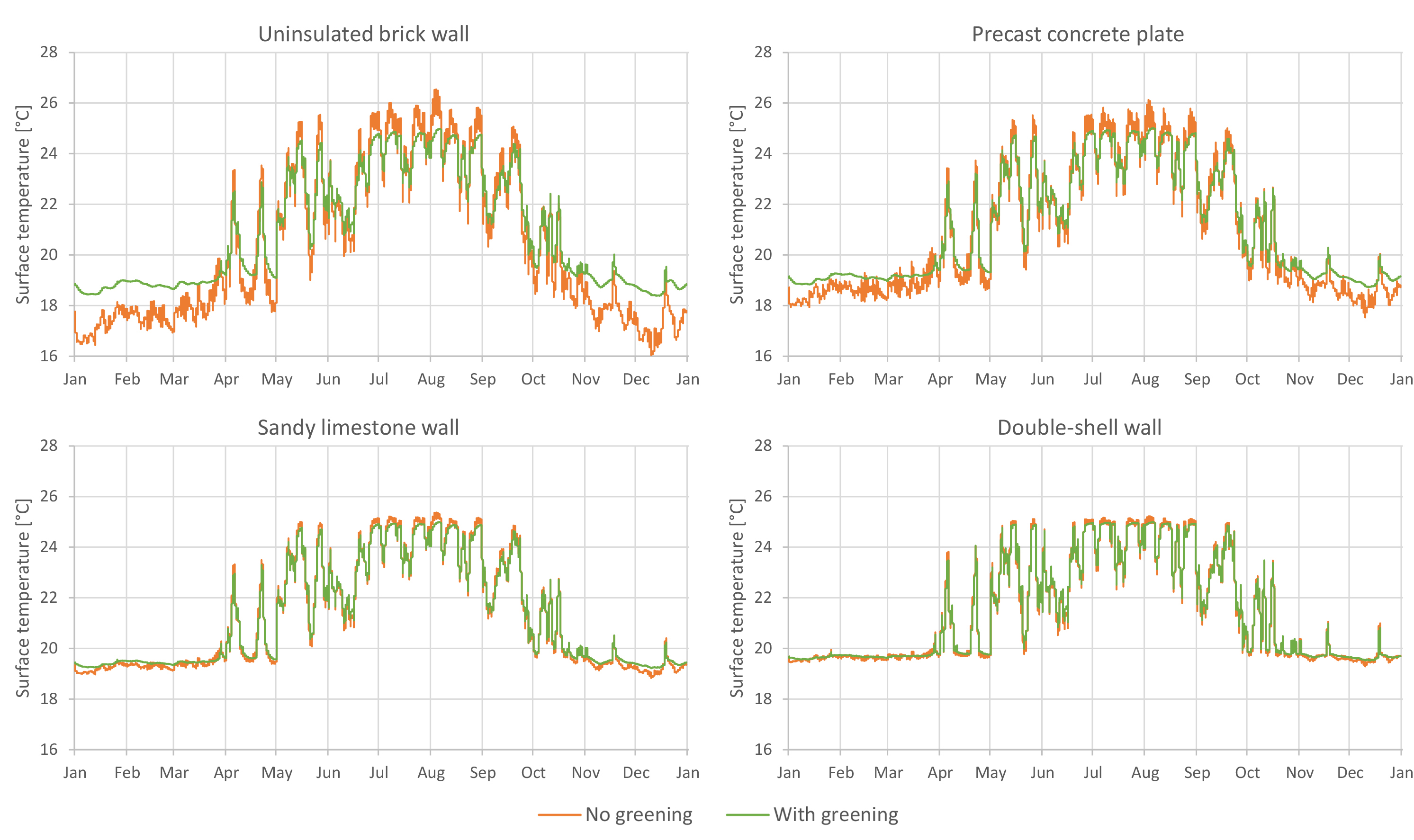

Supplement: Supplementary file 1 [file mmc1.zip › DataInBrief_Submission/Figure 2 The simulated interior surface temperature of the investigated wall assemblies.jpg]

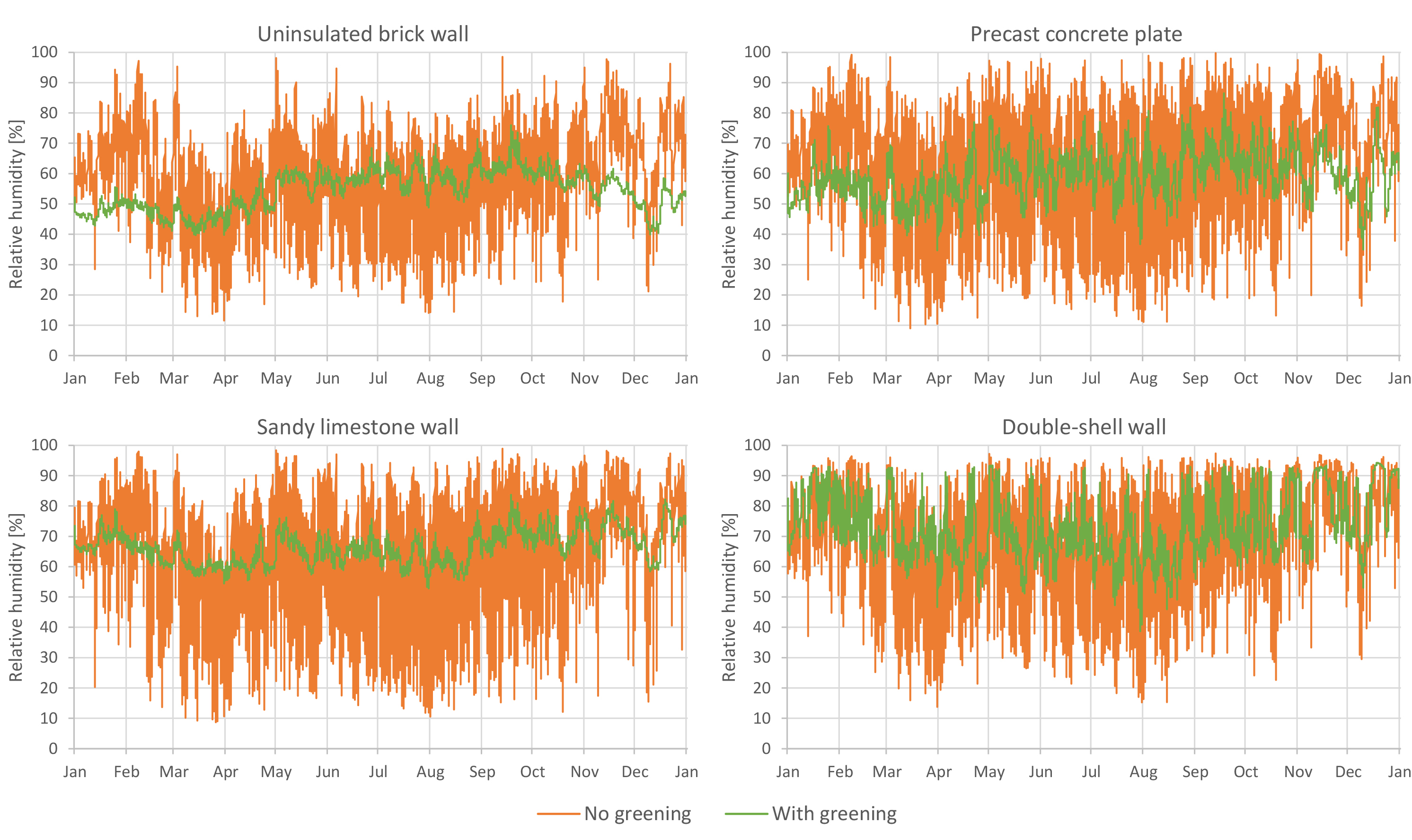

Supplement: Supplementary file 1 [file mmc1.zip › DataInBrief_Submission/Figure 3 The simulated relative humidity of the exterior surface of the investigated wall assemblies.jpg]

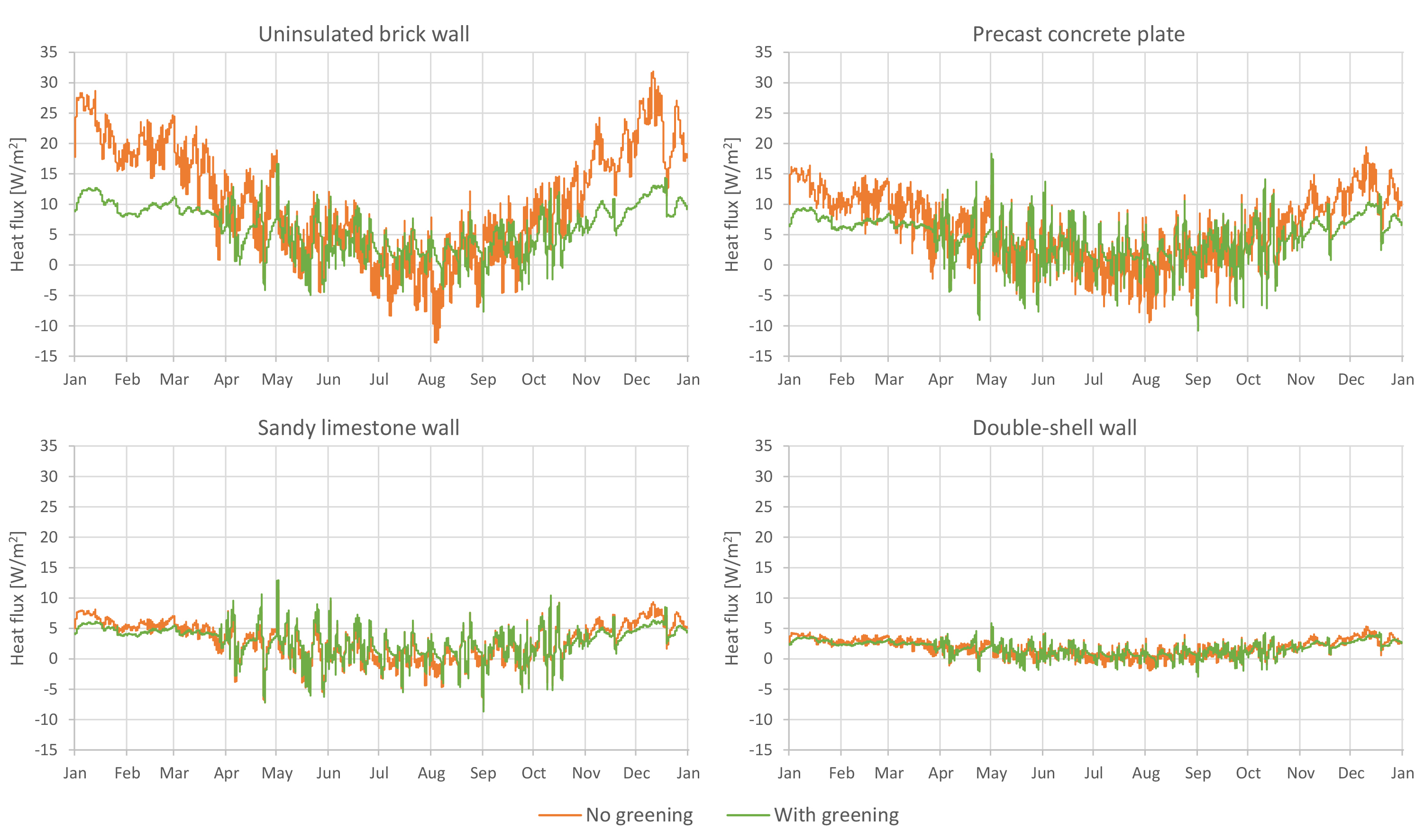

Supplement: Supplementary file 1 [file mmc1.zip › DataInBrief_Submission/Figure 4 The simulated heat flux through the investigated wall assemblies.jpg]

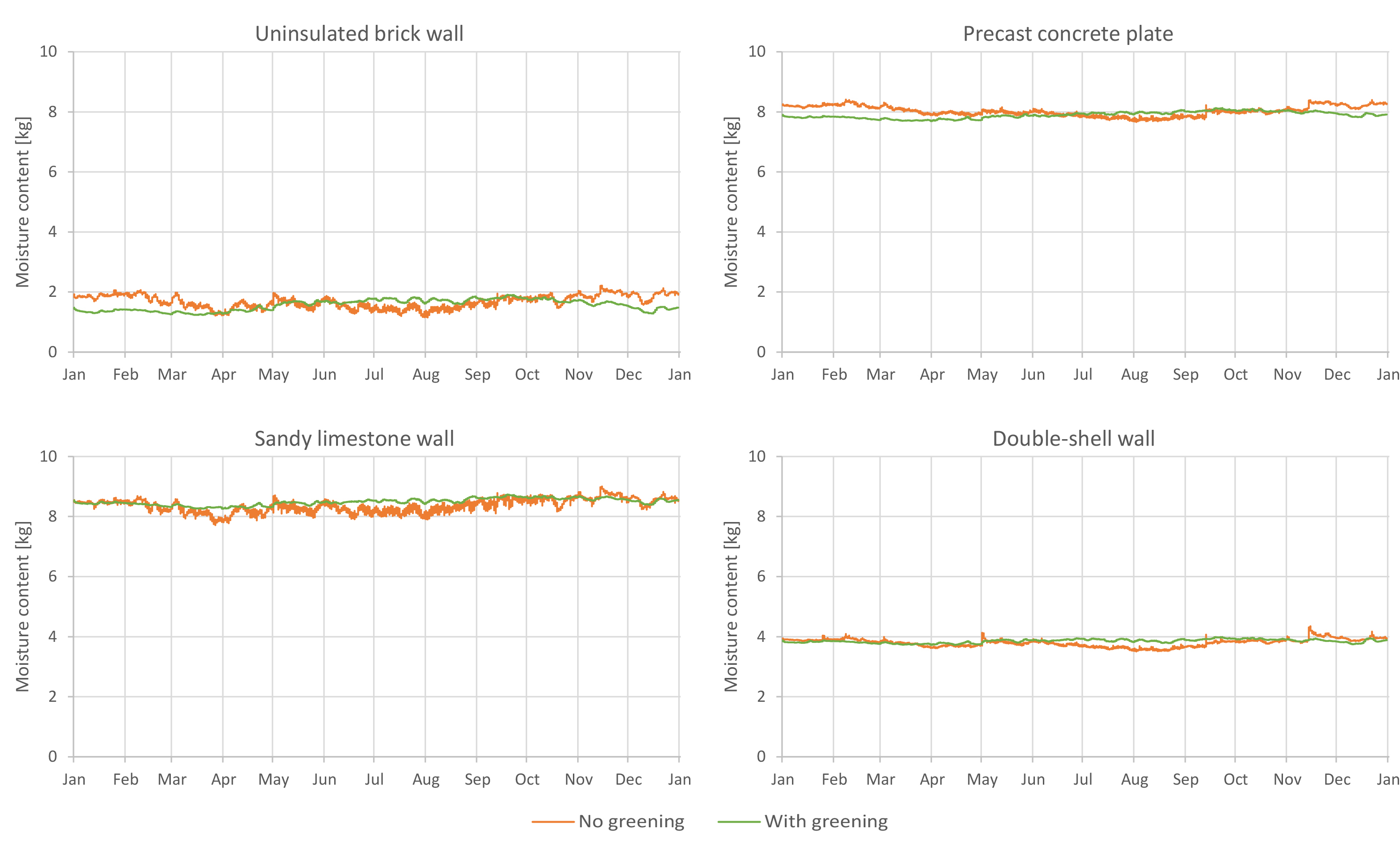

Supplement: Supplementary file 1 [file mmc1.zip › DataInBrief_Submission/Figure 5 The simulated moisture content in the investigated wall assemblies.jpg]

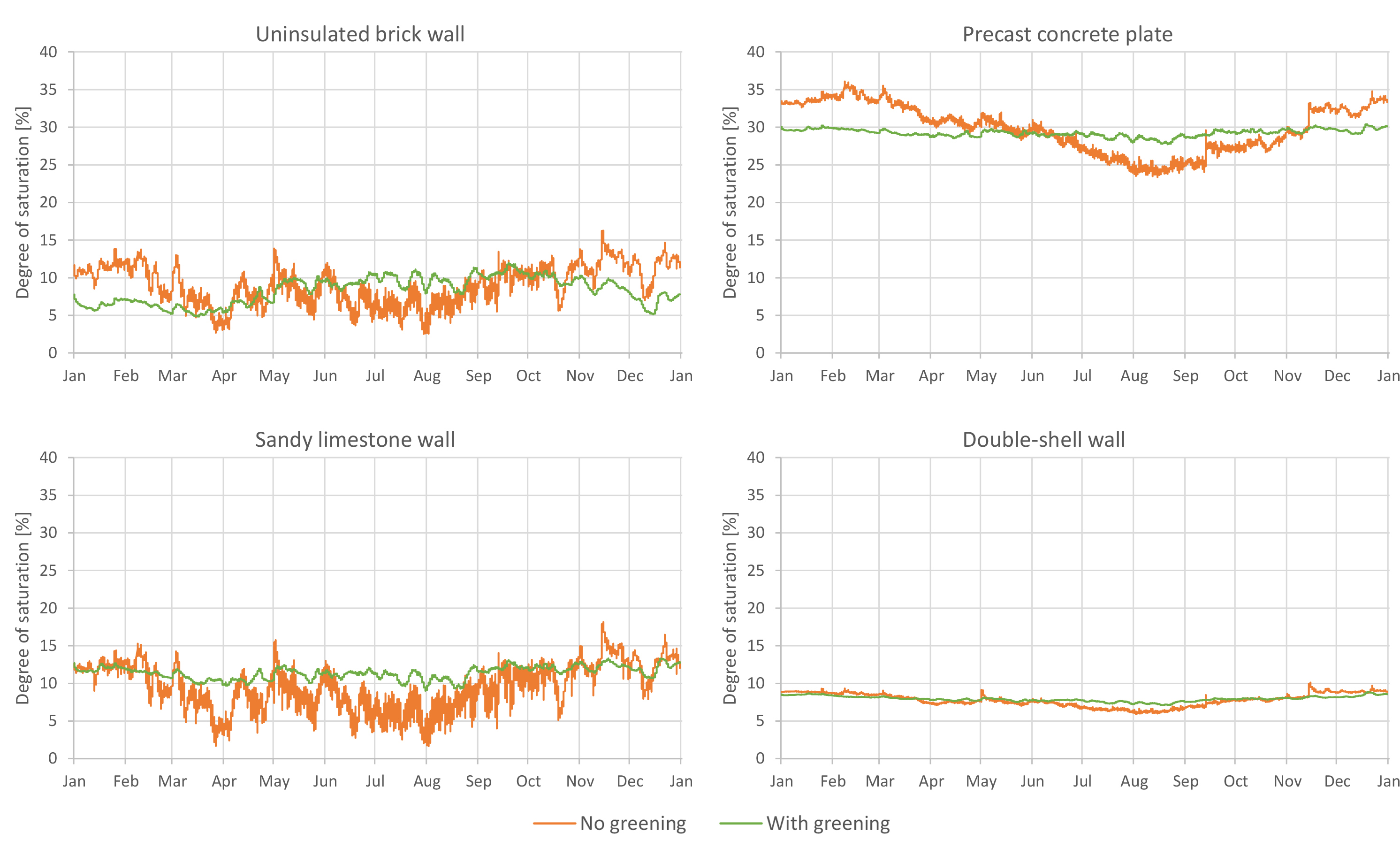

Supplement: Supplementary file 1 [file mmc1.zip › DataInBrief_Submission/Figure 6 The simulated degree of saturation at the exterior finishes of the investigated wall assemblies.jpg]

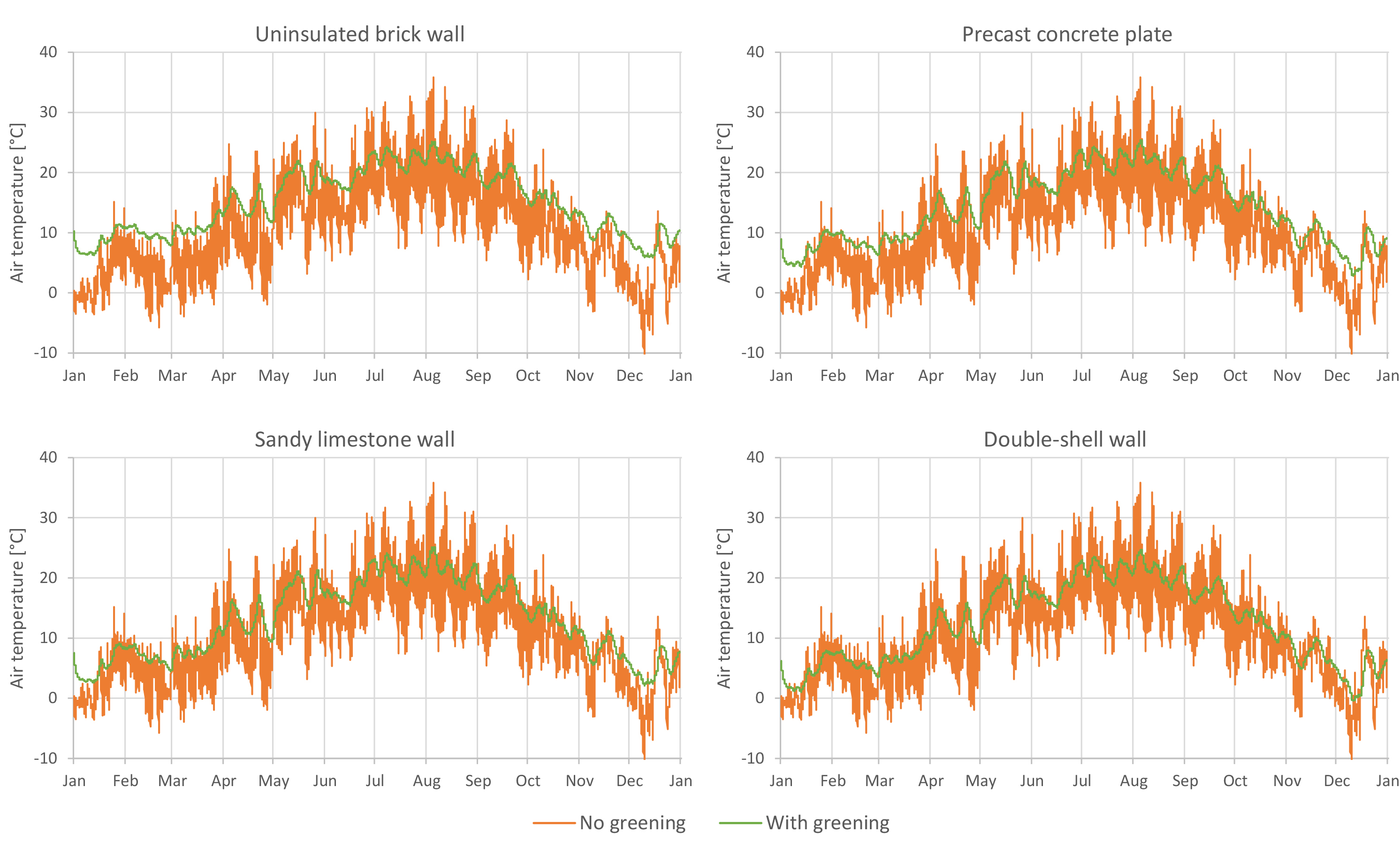

Supplement: Supplementary file 1 [file mmc1.zip › DataInBrief_Submission/Figure 7 The simulated air temperature adjacent to the wall.jpg]

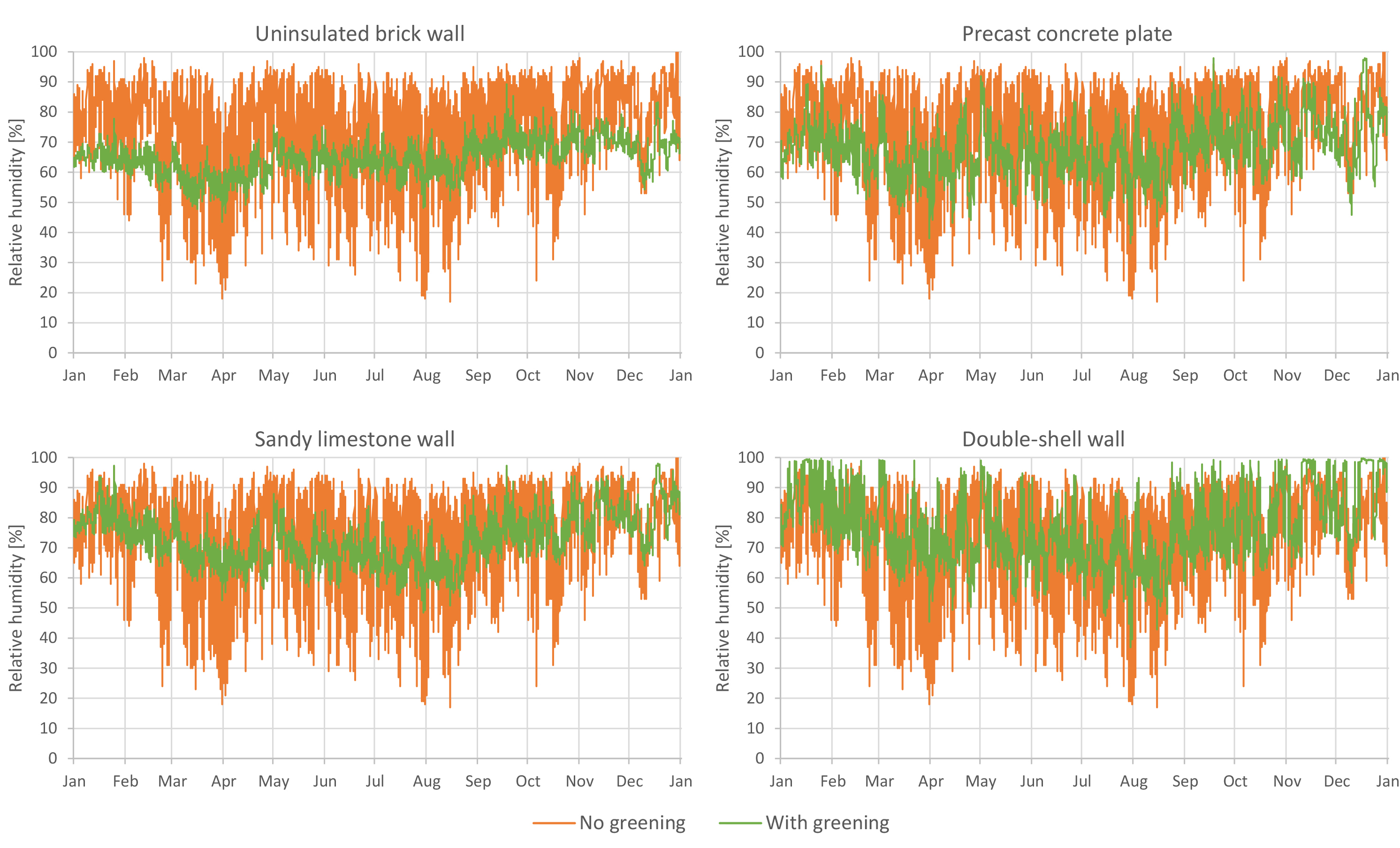

Supplement: Supplementary file 1 [file mmc1.zip › DataInBrief_Submission/Figure 8 The simulated relative humidity adjacent to the wall.jpg]

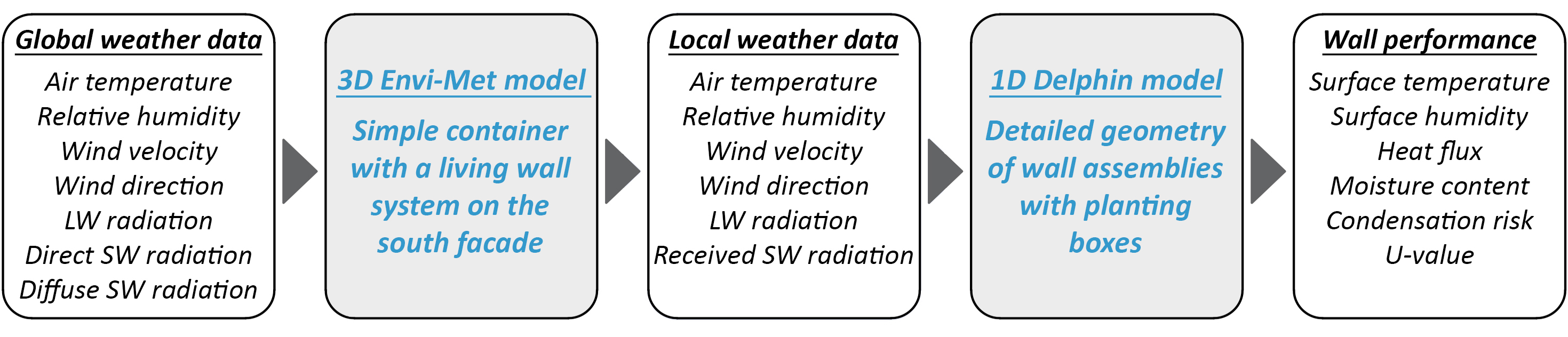

Supplement: Supplementary file 1 [file mmc1.zip › DataInBrief_Submission/Figure 9 The coupling of ENVI-Met and Delphin.jpg]
